# Supplementary material for: Establishing reference values for age-related fecal calprotectin in healthy children aged 0–4 years: a systematic review and meta-analysis
Source: PeerJ. 2025 Jun 12;13:e19572. doi: 10.7717/peerj.19572 (PMC12169166; doi:10.7717/peerj.19572)
Supplement: Supplemental Information 2 [file peerj-13-19572-s002.docx]

# Quality assessment

Table S1. Quality appraisal of the included studies using Joanna Briggs Institute Critical Appraisal Checklist

| **Source** | **JBI Critical Appraisal items*** | | | | | | | | | **Overall appraisal** | **Level*** |
| --- | --- | --- | --- | --- | --- | --- | --- | --- | --- | --- | --- |
|  | 1 | 2 | 3 | 4 | 5 | 6 | 7 | 8 | 9 |  |  |
| Marta, 2019[^22]^ | Y | Y | Y | Y | Y | Y | Y | Y | Y | Include | 1 |
| Campeotto,2003^[23]^ | Y | Y | U | Y | Y | Y | Y | Y | Y | Include | 2 |
| Li F, 2015^[5]^ | Y | Y | Y | Y | Y | Y | Y | Y | Y | Include | 1 |
| Hestvik, 2011^[24]^ | Y | Y | U | Y | Y | Y | Y | Y | Y | Include | 2 |
| Rugtveit, 2002^[25]^ | Y | Y | U | Y | Y | Y | Y | Y | Y | Include | 2 |
| Nissen, 2004^[26]^ | Y | Y | U | Y | Y | Y | Y | Y | Y | Include | 2 |
| Oord T, 2014^[4]^ | Y | Y | U | Y | Y | Y | Y | Y | Y | Include | 2 |
| Zhu Q, 2016^[3]^ | Y | Y | U | Y | Y | Y | Y | Y | Y | Include | 2 |
| Song J, 2016^[27]^ | Y | Y | U | Y | Y | Y | Y | Y | Y | Include | 2 |
| Macqueen,2018^[28]^ | Y | Y | Y | Y | Y | Y | Y | Y | Y | Include | 1 |
| Lee Y, 2017^[29]^ | Y | Y | Y | Y | Y | Y | Y | Y | Y | Include | 1 |
| Günaydın, 2020^[30]^ | Y | Y | Y | Y | Y | Y | Y | Y | Y | Include | 1 |
| Garg, 2017^[31]^ | Y | Y | U | Y | Y | Y | Y | Y | Y | Include | 2 |
| Park, 2020^[32]^ | Y | Y | U | Y | Y | Y | Y | Y | Y | Include | 2 |
| Rouge, 2010^[33]^ | Y | Y | U | Y | Y | Y | Y | Y | Y | Include | 2 |
| Roca, 2017^[34]^ | Y | Y | Y | Y | Y | Y | Y | Y | Y | Include | 1 |
| Łoniewska, 2020^[35]^ | Y | Y | Y | Y | Y | Y | Y | Y | Y | Include | 1 |
| Li F, 2014^[36]^ | Y | Y | U | Y | Y | Y | Y | Y | Y | Include | 2 |
| Campeotto, 2020^[37]^ | Y | Y | U | Y | Y | Y | Y | Y | Y | Include | 2 |
| Zoonen, 2019^[38]^ | Y | Y | Y | Y | Y | Y | Y | Y | Y | Include | 1 |
| Laforgia,2003^[39]^ | Y | Y | Y | Y | Y | Y | Y | Y | Y | Include | 1 |
| Jung,2020^[40]^ | Y | Y | Y | Y | Y | Y | Y | Y | Y | Include | 1 |
| Myjak,2017^[41]^ | Y | Y | U | Y | Y | Y | Y | Y | Y | Include | 2 |

* JBI Critical Appraisal Checklist: 1: was the sample frame appropriate to address the target population? 2: were study participants sampled in an appropriate way? 3: was the sample size adequate? 4: were the study subjects and the setting described in detail? 5: was the data analysis conducted with sufficient coverage of the identified sample? 6: were valid methods used for the identification of the condition? 7: was the condition measured in a standard, reliable way for all participants? 8: was there appropriate statistical analysis? 9: was the response rate adequate, and if not, was the low response rate managed appropriately?

**Level 1 represents studies that met all 9 criteria, level 2 represents studies that do not meet all 9 criteria in the JBI critical appraisal checklist
