# Supplementary figures and images for: Establishing reference values for age-related fecal calprotectin in healthy children aged 0–4 years: a systematic review and meta-analysis
Source: PeerJ. 2025 Jun 12;13:e19572. doi: 10.7717/peerj.19572 (PMC12169166; doi:10.7717/peerj.19572)

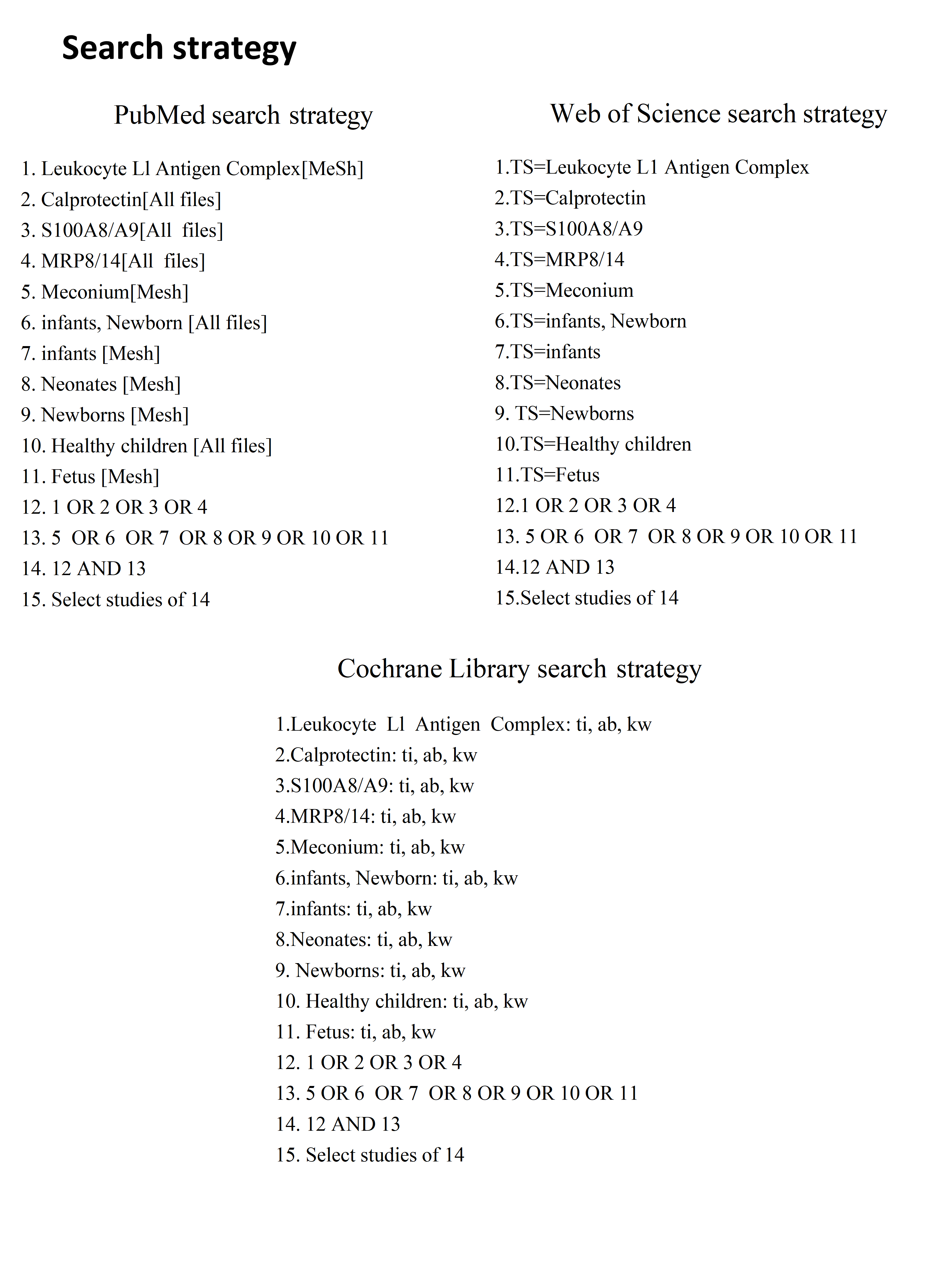

Supplement: Supplemental Information 3 [file peerj-13-19572-s003.png]

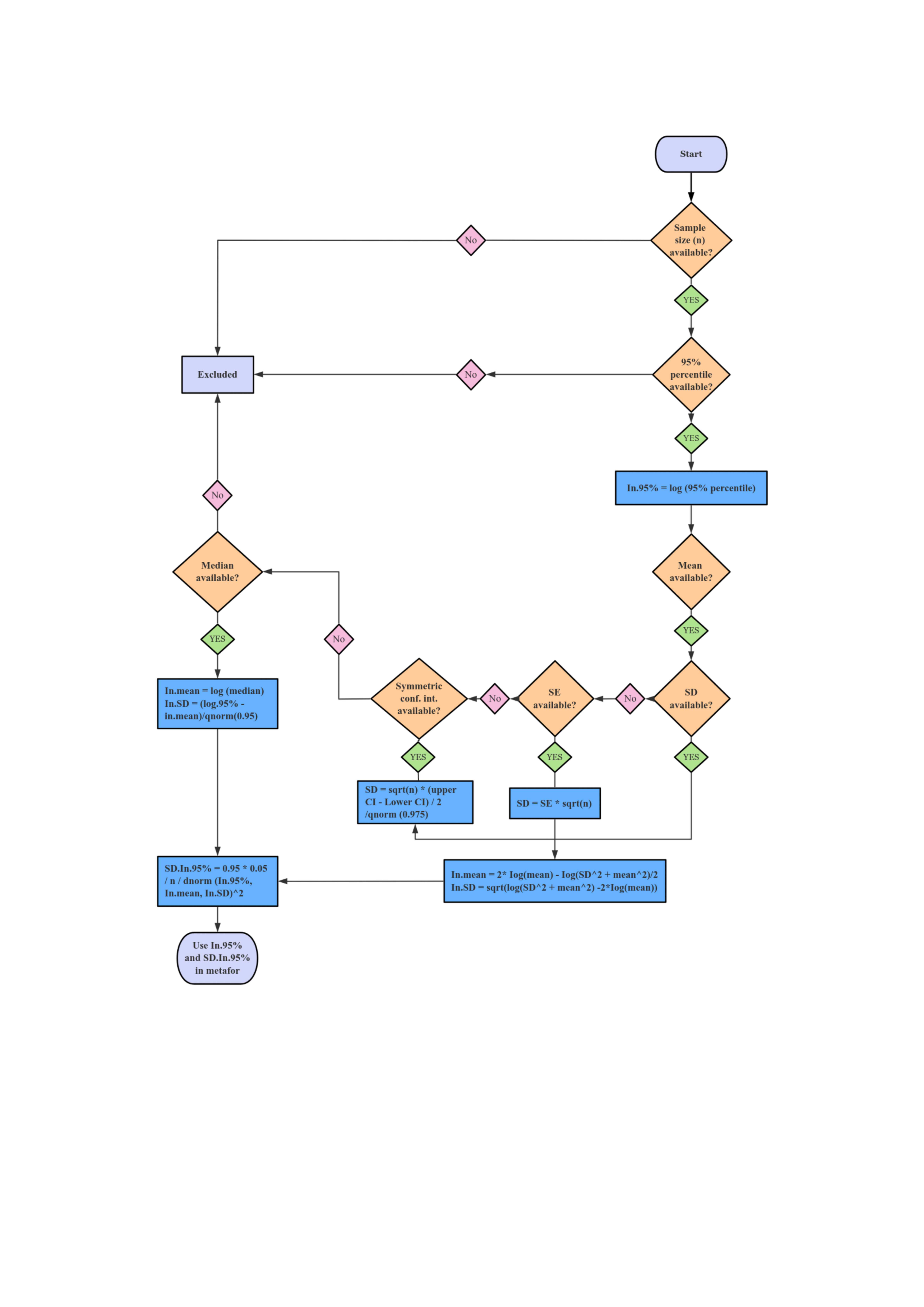

Supplement: Supplemental Information 4 [file peerj-13-19572-s004.png]

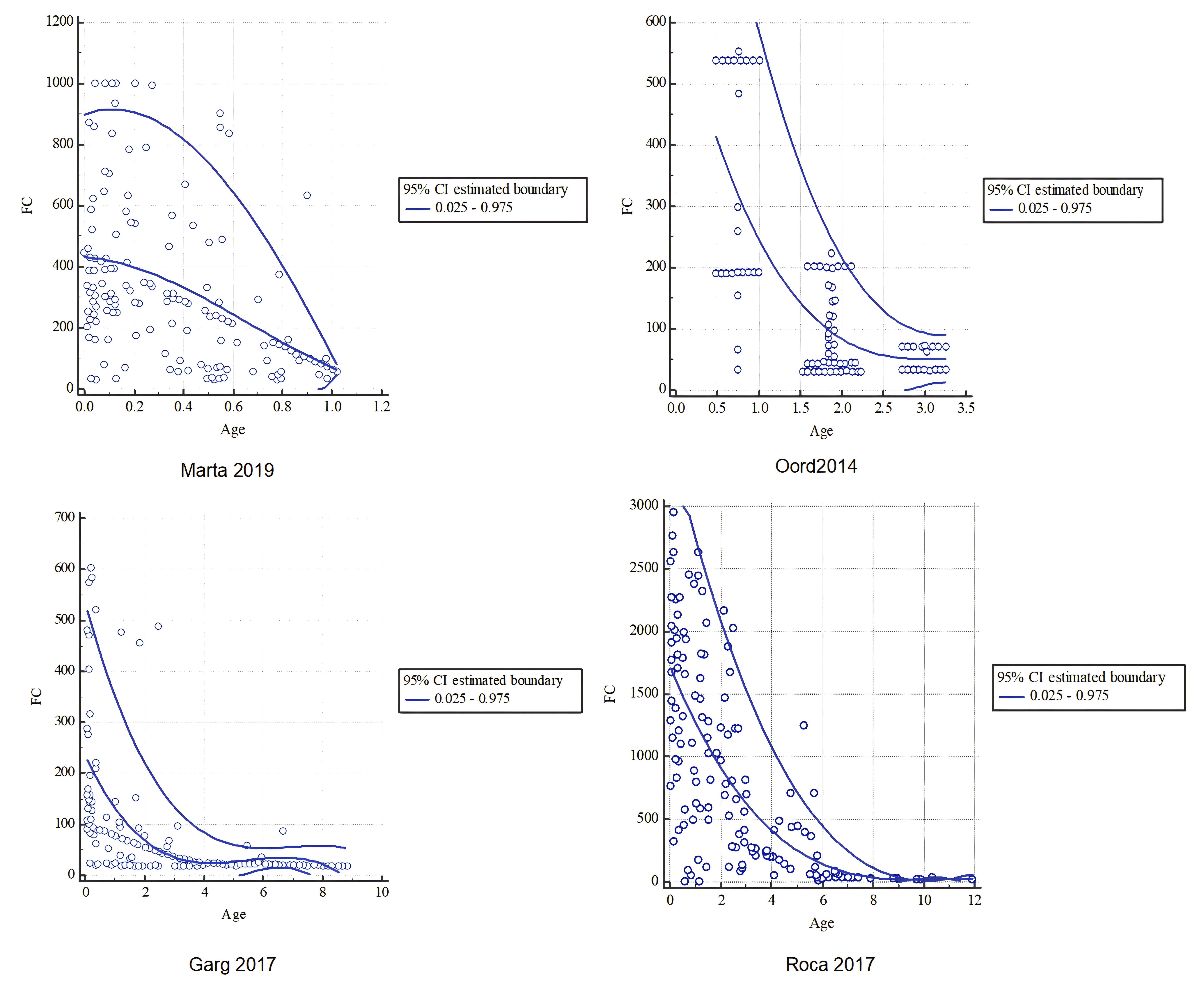

Supplement: Supplemental Information 5 — Fecal calprotectin values were extracted from graphical representations using the web-based tool “Web Plot Digitizer,” as the original data was presented in a visual format and subsequently converted into numerical values for analysis. [file peerj-13-19572-s005.png]

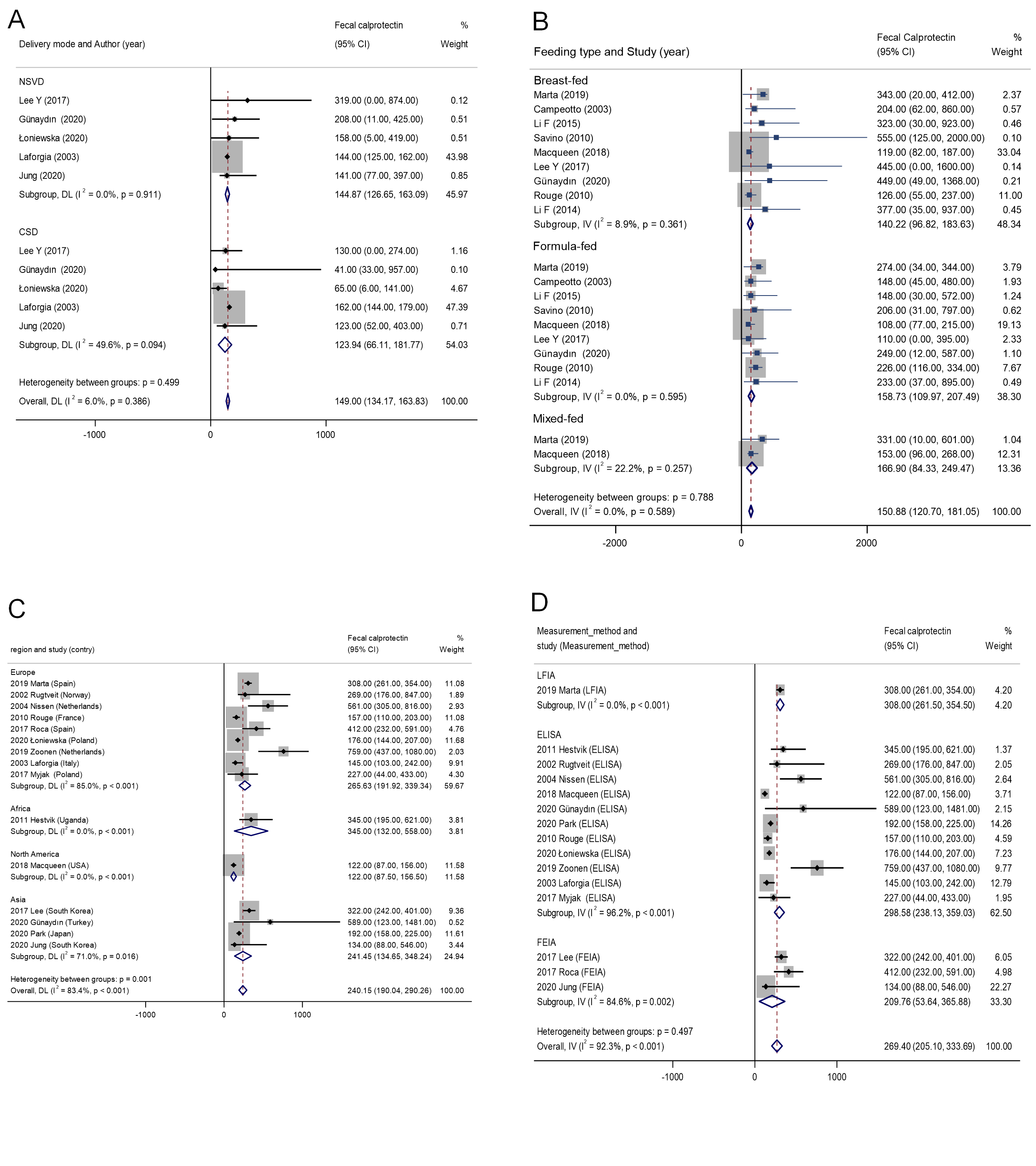

Supplement: Supplemental Information 6 — (A) Delivery mode, (B) feeding type, (C) geographical region, and (D) assay methodology. [file peerj-13-19572-s006.png]
