# Supplementary material for: Establishing reference values for age-related fecal calprotectin in healthy children aged 0–4 years: a systematic review and meta-analysis
Source: PeerJ. 2025 Jun 12;13:e19572. doi: 10.7717/peerj.19572 (PMC12169166; doi:10.7717/peerj.19572)
Supplement: Supplemental Information 8 [file peerj-13-19572-s008.docx]

The primary intended audience for this systematic review and meta-analysis is researchers and clinicians in the fields of pediatrics, neonatology, and gastroenterology. Researchers will utilize this review to understand the current state of evidence regarding age-related fecal calprotectin reference ranges in neonates, identify gaps in knowledge, and inform future research directions. Clinicians, particularly pediatricians and neonatologists, will find this review valuable for interpreting fecal calprotectin levels in neonates in clinical practice, potentially aiding in diagnosis and monitoring of neonatal gastrointestinal health.
